# Study of tribological behavior of Cu–MoS_2_ and Ag–MoS_2_ nanocomposite lubricants

**DOI:** 10.1186/s40064-016-1702-y

**Published:** 2016-01-22

**Authors:** V. An, E. Anisimov, V. Druzyanova, N. Burtsev, I. Shulepov, M. Khaskelberg

**Affiliations:** Laboratory 12, Institute of High Technology Physics, National Research Tomsk Polytechnic University, 30 Lenin Ave., Tomsk, Russia 634050; North-Eastern Federal University, Belinskogo 58, Yakutsk, Russia; Material Properties Measurements Centre, Institute of Physics and Technology, National Research Tomsk Polytechnic University, 30 Lenin Ave., Tomsk, Russia 634050

**Keywords:** Molybdenum disulfide, Cu and Ag nanoparticles, Friction coefficient

## Abstract

Tribological behavior of Cu–MoS_2_ and Ag–MoS_2_ nanocomposite lubricant was studied. Cu nanoparticles produced by electrical explosion of copper wires and Ag nanoparticles prepared by electrospark erosion were employed as metal cladding modifiers of MoS_2_ nanolamellar particles. The tribological tests showed Cu–MoS_2_ and Ag–MoS_2_ nanocomposite lubricants changed the friction coefficient of the initial grease and essentially improved its wear resistance.

## Background

Molybdenum and tungsten disulfides due to their anisotropic layered crystal structure are characterized by unique properties. These materials are good solid lubricants and antifriction additives to oil and greases (An and Irtegov 2014), moreover MoS_2_ is a promising material for lithium ion batteries (Wang et al. 2010). With respect to application of molybdenum and tungsten disulfides as lubricants, the synthesis and the appropriate state of dispersed materials or films play an important role. For improving tribological properties of MoS_2_ several methods are used: decreasing the particle size (Hu et al. 2010), creation of adaptive lubricants (Prasad et al. 2000), a composite mixture with other lubricants etc. As concerns composite lubricants, Sb_2_O_3_–MoS_2_ (Zabinski et al. 1993), Ag–MoS_2_ (Zhang et al. 2012), Ti–MoS_2_ (Renevier et al. 2001; Ilie and Tita 2007), Ni–WS_2_ (Wang et al. 2008) composites have shown a positive effect on tribological properties in comparison with pure compounds. Copper and copper alloys are well known lubricant materials due to the zero-wear friction effect discovering in 1956 (Garkunov 2000) and widely used in composite lubricants with molybdenum disulfide, especially for applications in vacuum (Kolesnichenko et al. 1986; Merstallinger et al. 2007; Kato et al. 2003). However, a synergetic effect of excellent antiwear properties of copper and antifriction behavior of MoS_2_ is observed in air at room temperature (An et al. 2014). The present paper is devoted to the study of the composition dependence on tribological properties of greases doped with Cu–MoS_2_ and Ag–MoS_2_ nanocomposites.

## Results and discussion

**Fig. 1:**
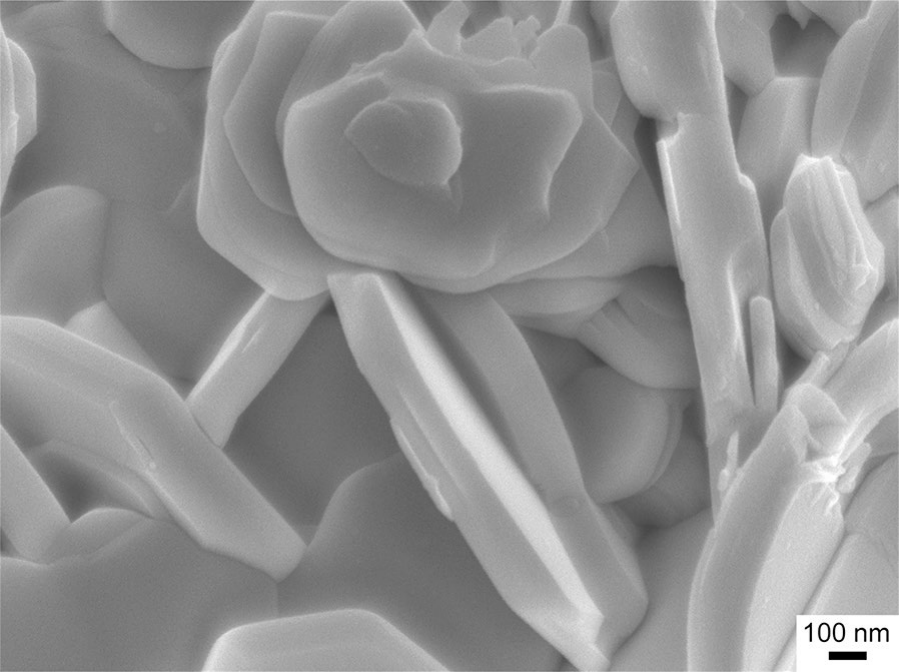
SEM image of undoped nanolamellar MoS_2_ produced by SHS

An SEM image of nanolamellar MoS_2_ (n-MoS_2_) produced by self-propagating high-temperature synthesis (SHS) from electroexplosive molybdenum nanopowders and pure elementary sulfur is presented in Fig. 1. The particles possess a layered hexagonal shape. According to the XRD data, the main phase in the final SHS products is 2H-MoS_2_. The prepared n-MoS_2_ particles mixed with n-Cu and n-Ag particles in different ratios were then added to the Litol and VNIINP greases. All samples were subjected to tribological tests.

**Fig. 2:**
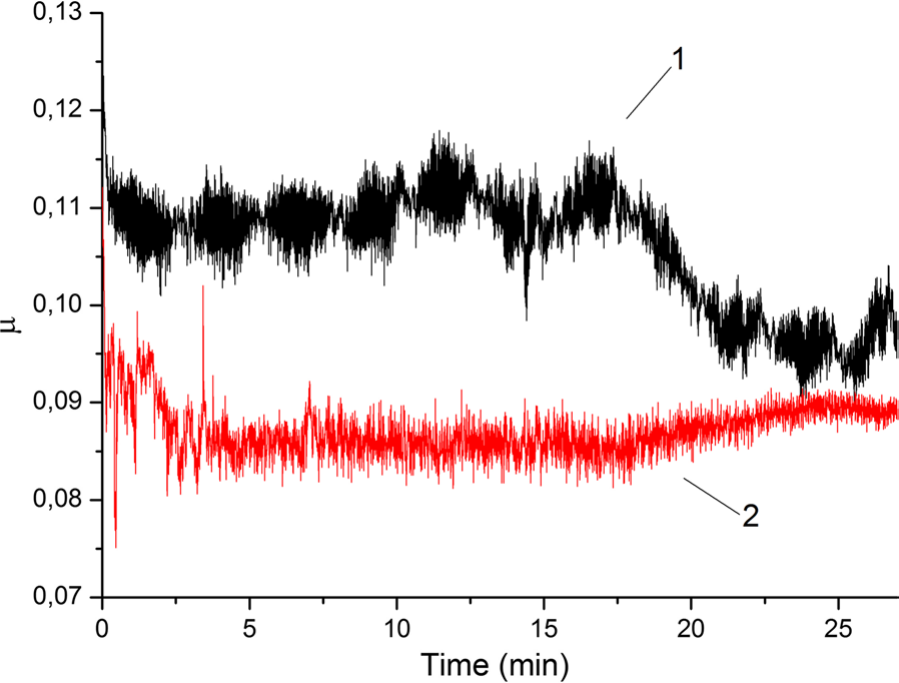
Friction coefficient of *1*—VNIINP grease, *2*−VNIINP grease + 5 % MoS_2_-05 + 7 % Cu)

Figure 2 shows the evolution of the friction coefficient versus time for the commercial grease VNIINP undoped and doped with composites of nanolamellar MoS_2_ and copper nanoparticles, 5 and 7 wt%, respectively. The doped grease reveals a lower average friction coefficient (μ_aver._ = 0.09) than that of undoped grease (μ_aver._ = 0.11). At the same time, doping the grease with the composition of nanolamellar MoS_2_ with n-Cu leads to a friction coefficient more stable in time. Apparently, this fact is related to the metal cladding effect caused by the presence of copper nanoparticles.

**Fig. 3:**
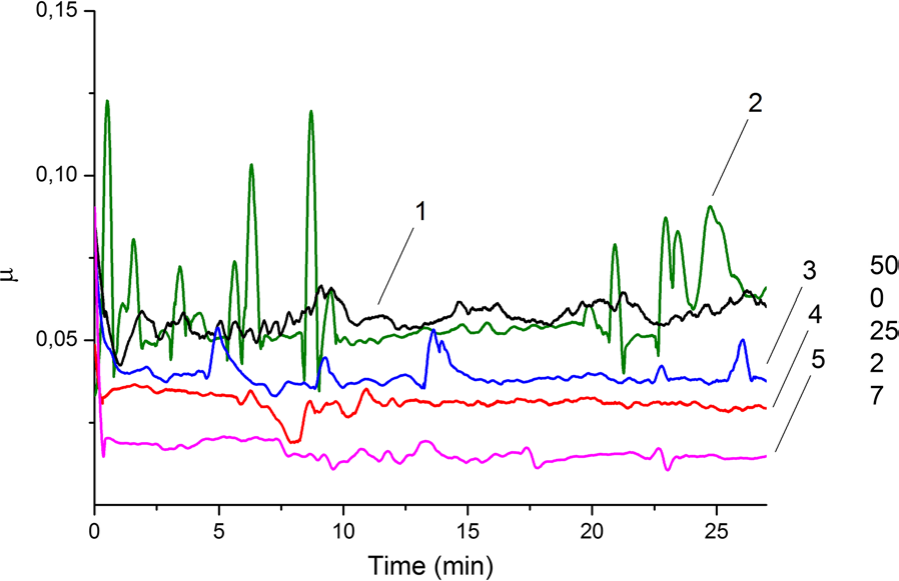
Friction coefficient of powders *1*—MoS_2_ + 50 % Cu, *2*—MoS_2_, *3*—MoS_2_ + 25 % Cu, *4*—MoS_2_ + 2 % Cu, *5*—MoS_2_ + 7 % Cu

Figure 3 displays the friction coefficient versus time for the n-MoS_2_ doped with n-Cu in different ratios: 2, 7, 25, and 50 wt% n-Cu. Surprisingly, the lowest average friction coefficient (μ_aver._ < 0.025) was found for n-MoS_2_ doped with 7 wt% n-Cu. It is lower than that of n-MoS_2_ doped with 2 wt% n-Cu (μ_aver._ ~ 0.027). It should be noted again that copper nanoparticles impact positively on the stability of the friction coefficient in time in comparison with undoped n-MoS_2_. The n-Cu particles clad wear fissures on the surface that leads to the formation of a soft tribofilm which allows n-MoS_2_ particles to slide on the copper tribofilm easier than on the steel disk surface. The formation of the tribofilm was verified by the AFM measurements.

**Fig. 4:**
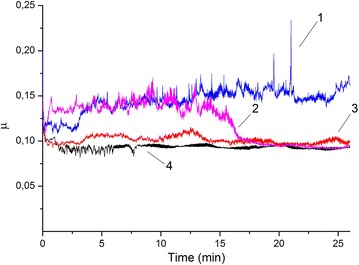
Friction coefficient of Grease *1*—Litol + 5 % n-MoS_2_, *2*—Litol, *3*—Litol + 5 % (MoS_2_ + 7 % Ag) *4*—Litol + 5 % (MoS_2_ + 7 % Cu)

The doped Litol grease showed remarkable results for the tribological tests (Fig. 4). The lowest friction coefficient (μ_aver._ ~ 0.09) was detected for the grease Litol doped with 5 % of the n-additive (n-MoS_2_ + 7 % n-Cu). This value is lower in comparison with the undoped Litol grease or doped with 5 % of n-MoS_2._

**Fig. 5:**
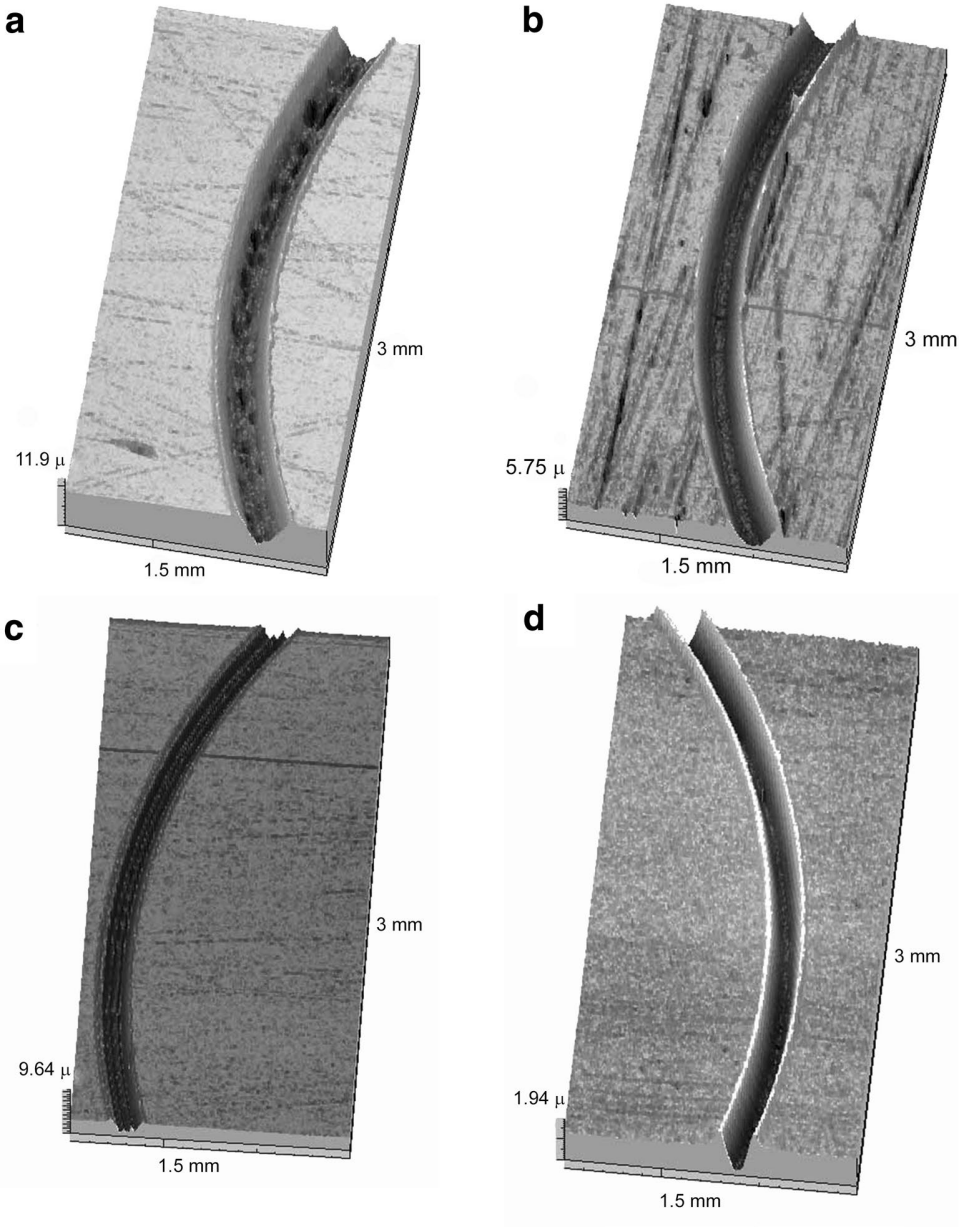
Wear tracks of the steel disk after the friction tests with **a** Litol + 5 % MoS_2_, **b** Litol, **c** Litol + 5 % (MoS_2_ + 7 % Ag) **d** Litol + 5 % (MoS_2_ + 7 % Cu)

Figure 5 and Table 1 illustrate appropriate antiwear properties of n-Cu and n-Ag additives to the Litol and VNIINP greases. 5 %-additives of n-MoS_2_ shows even an increase in wear which is apparently related to humidity conditions and a not-stable state of n-MoS_2_ in the grease. The presence of spikes in the wear tracks is probably related to random contacts between the disk and the ball. Nevertheless, n-MoS_2_ doped with n-Cu or n-Ag can reduce wear. In case of the VNIINP grease, wear can be even negative because of the ovecladding effect when copper nanoparticles “splice” the steel surface. The AFM measurements are also in good agreement with such an assumption.

**Table 1:** Wear and roughness of the steel disks after the friction tests

Sample	Wear (μm^3^ 10^−6^)	Roughness of the track (nm)
Litol grease	39.3128	35
Litol grease +5 % n-MoS_2_	172.53358	210
Litol grease + 5 % (n-MoS_2_ + 7 % n-Cu)	12.73898	32
Litol grease 5 % (n-MoS_2_ + 7 % n-Ag)	32.69368	93
VNIINP grease	15.15992	25
VNIINP grease + 5 % (n-MoS_2_ + 7 % n-Cu)	−2.17288	102

## Conclusions

Composite greases containing nanolamellar MoS_2_ doped with Cu and Ag nanoparticles were successfully prepared for tribological tests. The performed tribological tests showed the better antifriction performance for both the solid n-MoS_2_ lubricant doped with copper nanoparticles and the Litol and VNIINP greases doped with n-MoS_2_ with copper nanoparticles. Electroexplosive copper and electroerosive silver nanoparticles can improve the n-MoS_2_ tribological performance due to a visible rise in antiwear characteristics. At the same time, we can expect essential improvement of oxidation stability of the greases doped with the studied metal nanoparticles, especially with n-Ag. Another explanation for the improvement of the properties is related to a synergetic effect in using nanolamellar molybdenum disulfide and metal cladding additives of Cu and Ag nanoparticles.

## Experimental

MoS_2_ nanolamellar particles (n-MoS_2_) produced by self-propagating high-temperature synthesis (SHS), as well as copper (n-Cu) and silver nanoparticles (n-Ag) obtained by electrical explosion of wires (EEW) and electrospark erosion, respectively, were used for preparing a composite lubricant. SHS of metal sulfides from metal nanopowders is discussed in Irtegov et al. (2012). Conditions and parameters of electrical explosion of copper wires are presented in An et al. (2014). The initial powders were analyzed using an X-ray diffractometer Shimadzu XRD-7000 diffractometer (CuK_*α*_ irradiation) and a scanning electron microscope (JSM-7500FA, JEOL). In order to minimize agglomeration, the nanoparticles were subjected to ultrasonic treatment in an organic solvent before the preparation of the greases. For tribological tests MoS_2_ nanolamellar particles and Cu nanopowder are mechanically mixed during 30 min. Copper content in composite lubricant was 2, 7, 25 and 50 wt%, respectively. Besides, a solid lubricant, complex soap based greases (LITOL and VNIINP) with Cu–MoS_2_ additives were produced by dispersing using ultrasonic bath. Before dispersing, viscosity of greases was decreasing by addition of hexane. After dispersing composite greases are dried at room temperature during 24 h. Tribological investigations of pure nanolamellar MoS_2_, composite Cu–MoS_2_ lubricants and greases were carried out by “ball-on-disk” PC-Operated High Temperature Tribometer TXT-S-AH0000, CSEM. The wear scar was explored on a noncontact profilometer Micro Measure 3D Station, STIL. All tests were carried out using a 30 mm diameter medium-carbon steel disks as the friction body, and a vanadium-cobalt ball of diameter 3 mm was used as the counterface. The tests were run using a load of 5 N and sliding speed of 5 cm/s, with track diameter 3 mm, duration of tests was 30 min. The mean contact pressure was 0.56 N/mm^2^. After friction tests surface of wear scars were analyzed using an atomic force microscope Ntegra Aura (NT-MDT, Russia).
